# Perioperative management of severe factor VII deficiency: a single-center experience in China

**DOI:** 10.3389/fmed.2025.1673206

**Published:** 2025-09-22

**Authors:** Chao Zhu, Jinxia Zhao, Longqiang Xu, Wenfei Lu, Shiguo Liu, Jialiang Guan

**Affiliations:** ^1^Department of Clinical Laboratory, The Affiliated Hospital of Qingdao University, Qingdao, China; ^2^Department of Medical Genetics, The Affiliated Hospital of Qingdao University, Qingdao, China; ^3^Department of Emergency Internal Medicine, The Affiliated Hospital of Qingdao University, Qingdao, China

**Keywords:** factor VII deficiency, perioperative management, replacement therapy, prothrombin complex, antifibrinolytic agents

## Abstract

**Introduction:**

Inherited factor VII (FVII) deficiency is a rare autosomal recessive disorder whose clinical phenotypes are highly variable. Many studies have observed the absence of a clear-cut and consistent correlation between bleeding symptoms and FVII levels. Perioperative bleeding is a major concern in patients with FVII deficiency, but validated recommendations about the perioperative management of replacement therapy (RT) with FVII are lacking.

**Methods:**

Our study retrospectively summarized and analyzed the perioperative hemostasis management of severe FVII deficiency in 20 patients.

**Results:**

We found that replacement therapy is generally effective and that there is no significant correlation between the perioperative hemorrhagic complications after RT and the severity of FVII level before RT. Through multivariate statistical analysis and a retrospective analysis of other coagulation factor deficiencies at our center, we found that postoperative secondary hyperfibrinolysis in patients with FVII deficiency may not be universal.

**Discussion:**

Antifibrinolytic treatment may be necessary for patients undergoing surgery at sites with high fibrinolytic activity during the perioperative period. In addition, clinical data such as bleeding phenotype, bleeding history, and surgical sites should be given appropriate attention in perioperative treatment and monitoring.

## Introduction

Inherited factor VII (FVII) deficiency is a rare autosomal recessive disorder with an incidence of 1 per 300,000 to 500,000 individuals. Unlike hemophilia A and B, clinical phenotypes in patients with FVII deficiency are highly variable, ranging from asymptomatic presentations to life-threatening hemorrhages, such as those involving the central nervous system (CNS), gastrointestinal (GI), or joints. Many studies have reported a lack of a clear and consistent correlation between bleeding symptoms and FVII levels (1, 2). Asymptomatic adults with mild or moderate FVII deficiency usually do not require treatment to maintain physiological hemostasis, whereas severe FVII deficiency may increase the risk of bleeding, although this is not always the case (3–5). Treatment is primarily by replacement therapy. Patients with factor VII (FVII) deficiency can be treated by infusion of fresh-frozen plasma, plasma-derived FVII concentrates, prothrombin complex concentrates (PCC) or human prothrombin complex (PPSB), and low-dose recombinant activated FVII (rFVIIa). The antifibrinolytic agents have also been used as supplements for the replacement therapy in some cases. Clinical data suggest that a mild elevation of plasma FVII levels (>10% normal) results in improved hemostasis (6). Perioperative bleeding is a major concern in patients with FVII deficiency, but validated recommendations about the perioperative management of replacement therapy (RT) with FVII are lacking (7). In the current study, we retrospectively summarized and analyzed perioperative hemostatic management of severe FVII deficiency in 20 patients.

## Patients and methods

### Patients and surgical procedures

The records of 20 patients with severe FVII deficiency (FVII: C < 10%), including 4 males and 16 females, who underwent surgical operations at the Affiliated Hospital of Qingdao University from June 2019 to June 2024, were retrieved. The surgical procedures performed on these patients included obstetric (5 cases), gynecological (3 cases), orthopedic (2 cases), otolaryngological (2 cases), mammary (2 cases), thoracic (2 cases), hepatobiliary (1 case), thyroid (1 case), ophthalmic (1 case), and genitourinary (1 case) surgeries. We classified major surgical operations as those involving extensive resection requiring intervention in a body cavity, removal of an organ, or alteration of normal anatomy. Minor surgeries were considered to involve only the skin, mucus membranes, or connective tissue. According to this standard, all 20 patients underwent major surgical procedures.

Clinical and laboratory data, including coagulation function profile, type of surgery, RT (replacement treatment) dose and duration, and bleeding presentation, were recorded. The management of patients’ pre-surgical and post-surgical procedures and outcomes of hemostatic treatment and bleeding complications were analyzed.

### Coagulation function assay and bleeding risk assessment

The peripheral blood samples were collected via venipuncture and anticoagulated with 0.038% citric acid. Coagulation function was screened using activated partial thrombin time (APTT) and prothrombin time (PT). The activity of coagulation factors was determined by either APTT- or PT-based assay with specific coagulation factor-depleted plasma. Severe FVII deficiency is defined by levels < 0.1 IU/mL (FVII: C < 10%), whereas partial deficiency is defined by levels between 0.2 IU/mL and 0.1 IU/mL (8, 9). Patients with FVII: C below 0.1 IU/mL and a personal history of bleeding, combined with another coagulopathy, major surgery, or surgery comprising the oropharyngeal or involving genitourinary mucosa or other organs in high fibrinolytic activity, were considered to be at higher risk of bleeding for surgical operations (10, 11).

### Therapeutic regimen

Human prothrombin complex (PPSB) or recombinant activated factor VII (rFVIIa) were used as replacement agents in the current study. The target level of FVII: C level for each patient was determined by the type and site of the surgical procedure, taking into account the patients’ bleeding history. For the majority of patients, an FVII: C level of 20% was considered to be compatible with the hemostatic requirements of operations, except for procedures involving organs with high fibrinolytic activity, where levels needed to be increased to 30% (11). However, some studies suggested that a mild elevation of plasma FVII levels (>10% normal) may improve hemostasis. The majority of patients were routinely given a transfusion once every 6 h for 3 consecutive days, i.e., operation day and a day pre-operation and post-operation, to achieve the desired FVII: C level (Table 1). The PT test, or activity of FVII, seems to be an optional way to monitor treatments in FVII deficiencies. Additional treatment was administered to patients with prolonged bleeding complications after surgery. For some patients whose procedures involved high fibrinolytic positions or hyperfibrinolysis post-operation, the antifibrinolytic therapy was conducted with aminomethylbenzoic acid (PAMBA) (0.6 g per day) during operation and days post it along with replacement therapy.

**Table 1:** Perioperative treatment and clinical data in 20 patients with severe FVII deficiency.

Patient number	Basic level of FVII: C (%) PT(s)		Treatments (PPSB/rFVIIa)	Therapeutic dose(IU/kg) Pre-operation Post-operation		Durations (days)	Peak level of FVII: C(%) PT(s)		Bleeding In-operation	PAMBA (0.6 g/d)
1	0.6	51.2	PPSB	18.0 IU/kg	18.0 IU/kg	q6h*2d	17.5	24.1	No	No
2	0.7	50.6	PPSB	16.8 IU/kg	16.8 IU/kg	q6h*2d	11.2	20.0	No	No
3	0.8	48.1	PPSB	28.8 IU/kg	23.1 IU/kg	q6h*3d	55.1	18.0	No	Yes
4	0.8	31.1	rFVIIa	25.64ug/kg	25.64 ug/kg	q6h*3d	/	8.0	No	No
5	0.9	43.2	PPSB	45.3 IU/kg	28.3 IU/kg	q6h*3d	65.6	10.7	No	No
6	1.0	45.7	PPSB	42.6 IU/kg	19.1 IU/kg	q6h*3d	45.3	12.2	No	Yes
7	1.0	66.7	PPSB	28.1 IU/kg	14.1 IU/kg	q6h*3d	51.3	17.1	No	No
8	1.3	42,3	PPSB	22.6 IU/kg	16.9 IU/kg	q6h*2d	10.0	/	No	Yes
9	1.5	37.0	PPSB	25.9 IU/kg	10.3 IU/kg	q6h*3d	38.2	/	No	No
10	1.6	39.2	PPSB	24.3 IU/kg	24.3 IU/kg	q6h*2d	46.1	30.0	No	No
11	1.9	33.5	PPSB	31.3 IU/kg	13.4 IU/kg	q6h*3d	59.5	/	No	Yes
12	1.9	38.0	PPSB	30.6 IU/kg	21.4 IU/kg	q6h*3d	23.6	/	No	No
13	2.0	35.2	PPSB	26.9 IU/kg	13.4 IU/kg	q6h*3d	46.5	15.9	No	No
14	2.4	37.6	PPSB	44.1 IU/kg	22.1 IU/kg	q6h*3d	64.7	/	No	No
15	3.1	25.1	PPSB	12.7 IU/kg	12.7 IU/kg	q6h*2d	25.7	18.6	No	No
16	3.6	39.8	PPSB	28.3 IU/kg	11.3 IU/kg	q6h*2d	40.6	16.3	No	No
17	3.8	29.7	PPSB	35.7 IU/kg	26.8 IU/kg	q6h*3d	62.3	/	No	No
18	5.6	31.7	PPSB	31.6 IU/kg	31.6 IU/kg	q6h*2d	54.2	/	No	No
19	6.6	32.6	PPSB	22.2 IU/kg	11.1 IU/kg	q6h*2d	37.9	14.3	No	No
20	8.4	28.3	PPSB	31.3 IU/kg	21.9 IU/kg	q6h*2d	55.6	17.0	Yes	No

PPSB, human prothrombin complex; PAMBA, aminomethylbenzoic acid; normal reference interval of FVII: C was 50.0–150.0%; normal reference interval of PT was 10.0–16.0 s.

The extent of bleeding during and after surgery was assessed to evaluate the outcome and efficacy of the hemostatic treatment. Patients who experienced greater-than-expected intraoperative and postoperative blood loss, excessive drainage, or significantly reduced hemoglobin levels requiring transfusion support were considered an excessive bleeding manifestation.

## Results

### Patient characteristics

The average age of the patients was 38.4 years, with an age range of 18 ~ 71 years, and the average weight was 63.5 kg (47.0 ~ 98.0 kg). The majority of patients were diagnosed incidentally during routine coagulation function screening for surgical/invasive procedures.

The average FVII: C of 20 patients was 2.5% (range 0.6 ~ 8.4%), including 5 cases (FVII: C ≤ 1%), 12 cases (FVII: C 1 ~ 5%), and 3 cases (FVII: C < 5 ≤ 10%). None of the patients had an inhibitor against FVII presented and none had a combined deficiency of other coagulation factors.

In total, 10(50.0%)patients had suffered episodes of bleeding, including 8 cases of menorrhagia, 1 case of gum bleeding, and 1 case of mucocutaneous bleeding. Overall, 11(55.0%)patients had undertaken surgical operations previously, and 7(35.0%)patients had received therapeutic transfusions of blood products (Table 1).

### Bleeding manifestations and hemostatic treatment outcomes

The preoperative average peak FVII: C achieved in 20 patients with replacement therapy was 42.68%, ranging from 10.0 to 64.7%, which mostly had prolonged PT corrected to the normal reference range (10.0 ~ 16.0 s). In total, 4 (20.0%) patients had received PPSB transfusion and antifibrinolytic therapy, while 15 (75.0%) patients only had PPSB transfusion, and 1 (5.0%) patient only had rFVIIa. Overall, 19 (95.0%) patients did not encounter excessive bleeding events, with less than 10 mL to 100 mL blood loss during operations (Table 1).

One (5.0%) patient suffered more than the usual expected bleeding, with 400 mL of blood loss intraoperatively, but not fatal. Although preoperative transfusion brought the patient’s PT to the normal reference range and FVII: C beyond 40%, the treatment failed to satisfy the hemostatic challenge of poor uterine contraction after delivery. In total, 3 (15.0%) patients suffered secondary hyperfibrinolysis post-operation, and conditions improved after antifibrinolysis treatment (Table 2). These three patients received orthopedic, otolaryngologic, and thoracic surgery, respectively, and the RT treatment achieved the expected results. We divided the 20 patients into 3 groups according to the basic FVII levels (FVII: C ≤ 1%; FVII: C:1 ~ 5%, and FVII: C < 5 ≤ 10%) before RT and included the statistical analysis between perioperative hemostasis complications and the FVII levels. The chi-square test showed that there was no significant correlation between perioperative hemostasis complications and FVII activity levels (χ2 = 1.771, *p* = 0.413).

**Table 2:** Data of four patients with hemorrhage in-operation or complications post-operation.

Patient	Bleeding history	PAMBA pre-operation	Fg (g/L)	FDP (mg/L)	D-D (mg/L)	Type of operation	Complications In-operation After-surgery		Treatment
Patient 7	Y	N	0.5	253.2	67.2	ankle	N	hyperfibrinolysis	Fg + FFP + CRYO
Patient 10	N	N	1.0	91.7	40.0	nasal	N	hyperfibrinolysis	Fg + PPSB
Patient 17	N	N	0.8	579.1	40.0	lungs	N	hyperfibrinolysis	Fg + FFP + CRYO
Patient 20	Y	N	3.4	49.6	25.1	cesarean	Bleeding	N	Oxytocin+PPSB

Fg, fibrinogen; D-D, D-Dimer; CRYO, cryoprecipitate; PAMBA, aminomethylbenzoic acid; normal reference interval of Fg was 1.8–3.5 g/L; normal reference interval of FDP was 0–5.0 mg/L; normal reference interval of D-D was less than 0.55 mg/L.

In order to prove that hyperfibrinolysis after surgery is not accidental, we also reviewed clinical data of 21 patients diagnosed with congenital coagulation factor (FII, FIX, and FX) deficiency in our center within the same period. All patients received PPSB transfusion as perioperative hemostasis treatment, and no such obvious secondary hyperfibrinolysis was found (Table 3). Meanwhile, we conducted a multivariate analysis between postoperative secondary hyperfibrinolysis and FVII level degree (based on the classification standard of 20% hemostatic activity level) after RT, bleeding history, surgical site, and trauma (high fibrinolytic activity/non-high fibrinolytic activity). Through the stratified Fisher exact test, it showed that the surgical site with high fibrinolysis activity had a higher risk of postoperative secondary hyperfibrinolysis without antifibrinolytic treatment (*p* = 0.036).

**Table 3:** Demographic and clinical characteristics of patients with FVII and F(II/IX/X) deficiency.

Characteristics	FVII deficiency (*N* = 20)	FII deficiency (*N* = 1)	FIX deficiency (*N* = 17)	FX deficiency (*N* = 3)
Age (yr)	38.4 ± 15.0	31	35.6 ± 15.3	38.7 ± 18.5
Sex (F/M)	16/4	1/0	0/17	3/0
Weight (kg)	63.5 ± 14.3	73.5	62.2 ± 11.0	76.0 ± 21.6
Bleeding history (%)	10/20(50.0)	0/1(0)	15/17(88.2)	2/3(66.7)
Transfusion history (%)	7/20(35.0)	1/1(100.0)	11/17(64.7)	2/3(66.7)
Family history (%)	1/20(5.0)	1/1(100.0)	3/17(17.6)	2/3(66.7)
Factor basic level (%)	2.48 ± 2.16	2.6	3.97 ± 2.63	9.93 ± 9.19
Replacement treatment	PPSB/rFVIIa	PPSB	PPSB	PPSB
RT dose (IU/kg)	28.8 ± 8.8/25.6ug/kg	24.5	45.9 ± 11.1	16.98 ± 14.74
RT interval(hours)	Per 6 h	Per 24 h	Per 24 h	Per 24 h
RT duration (days)	2.55 ± 0.51	2	11.47 ± 3.61	3.00 ± 0.00
Surgical operation				
Major	20	1	17	3
Minor	0	0	0	0
Antifibrinolytic therapy (%)	4/20(20.0)	0/1(0)	1/17(5.9)	0/3(0)
Bleedings (%)	1/20(5.0)	1/1(100.0)	6/17(35.3)	1/3(33.3)
Hyperfibrinolysis (%)	3/20(15.0)	0/1(0)	0/17(0)	0/3(0)

## Discussion

FVII plays a leading role in initiating the coagulation cascade *in vivo*. The extrinsic pathway is initiated at tissue damage sites, following the formation of an equimolar (1:1) complex between activated FVII (FVIIa) and tissue factor (TF). Activated FVII (FVIIa) normally circulates in minimal amounts (approximately 1% of the total FVII mass), which are in a steady-state condition with the FVII zymogen. Once FVII zymogen binds to TF, it will result in a cleavage at position 152 in the FVII sequence, thus creating an amount of FVIIa. The TF/FVIIa complex is a powerful activator of coagulation in both intrinsic and extrinsic pathways by activating larger amounts of FVII, factor IX (FIX), and FX (1, 12, 13). Congenital deficiency of FVII is characterized by a substantial decrease in the total FVII quantity, resulting in very low levels of circulating FVIIa, which is almost absent when measured with classical assays. Bleeding in patients with inherited FVII deficiency is extremely heterogeneous concerning both sites and severity. As a result, the minimal FVII levels avoiding bleeding in different clinical situations are still unknown and standardized elements influencing the clinical picture are uncharacterized up to now.

Bleeding during and after surgery is a potential complication for any surgical intervention and a major concern for the surgeon, so its control is essential for the successful outcome of surgery. The mainstay for bleeding prevention is replacement therapy (RT), based on the substitution of the missing factor, in order to correct the clotting defect (14). In our study, the perioperative management of the majority of patients was effective in producing competent hemostasis, except for one case with perioperative bleeding. Worth noting, the patient experiencing bleeding complications was in the highest level of basic FVII: C (8.4%) of 20 patients. We have reason to predict that the severity of FVII deficiency could not be predictive for the bleeding tendency excellently, while clinical data such as bleeding phenotype appeared to be predictive parameters of bleeding risk appropriately, similar to previous literature data (3, 7, 15). The bleeding history prompted the need for replacement therapy prior to surgery and meant a high risk of complications intraoperatively or postoperatively (16, 17). We also found that 3 (15.0%) patients suffered secondary hyperfibrinolysis post-operation. We divided the 20 patients into 3 groups according to the basic FVII levels (FVII: C ≤ 1%; FVII: C:1–5%, and FVII: C < 5 ≤ 10%) before RT and included the statistical analysis between perioperative hemostasis complications (perioperative bleeding and postoperative hyperfibrinolysis) and the basic FVII levels. The chi-square test showed that there was no significant correlation between perioperative hemostasis complications and FVII activity levels (χ2 = 1.771, *p* = 0.413).

In order to prove that hyperfibrinolysis after surgery is not universal, we specially reviewed clinical data of 21 patients diagnosed with congenital coagulation factor (FII, FIX, and FX) deficiency in our center within the same period. All patients received PPSB transfusion as perioperative hemostasis treatment, and no such obvious secondary hyperfibrinolysis was found. Meanwhile, we found that patients who were undergoing surgeries with high fibrinolysis activity had a higher risk of postoperative secondary hyperfibrinolysis without antifibrinolytic treatment through multivariate analysis by the stratified Fisher exact test (*p* = 0.036). Therefore, we have reason to predict that this phenomenon is not accidental, and for the procedures located in surgical areas with high fibrinolytic activity, the use of antifibrinolytic drugs might contribute to the good management of these types of surgery (18, 19). From our experience, the hemorrhagic or fibrinolytic tendency can only be treated by giving antifibrinolytics before giving fibrinogen/other concentrate; if these are not available, cryoprecipitate has the same similar effect, but we have insufficient controlled data to confirm this view.

While hyperfibrinolysis in each patient with FVII deficiency cannot be predicted reliably, some reports considered it appeared to be linked to the severity of the trauma and the organ systems affected (e.g., head injury and urogenital tract injury). Activation of the coagulation system leads to the simultaneous release of tissue plasminogen activator (t-PA) and its antagonist, plasminogen activator inhibitor type 1 (PAI-1). Initially, the increase in t-PA appears to outstrip that in PAI-1. In other studies, factor seven activating protease (FSAP) is a trigger of both coagulation and fibrinolysis, playing a key role in hemostasis and thrombosis. FSAP could activate FVII and scuPA (single-chain urokinase plasminogen activator) and other substrates, including fibrinogen and HMWK (high-molecular-weight kininogen), but it was a poor FVII activator (20, 21). We suspect a compensatory increase of FSAP in patients with FVII deficiency compared with healthy individualsuals, while further experimental evidence is needed to support this opinion.

## Conclusion

Our research found that replacement therapy is generally effective in perioperative management of severe factor VII deficiency, and there is no significant correlation between the perioperative hemorrhagic complications after RT and the severity of FVII level before RT. However, several patients with a history of bleeding need special attention for the risk of perioperative bleeding. Through multivariate statistical analysis and the retrospective study of other coagulation factor deficiencies in our center, we found that postoperative secondary hyperfibrinolysis in patients with FVII deficiency is not universal. It is necessary to give the treatment of antifibrinolytics for patients undergoing surgeries in high fibrinolytic sites during the perioperative period. Hyperfibrinolysis in patients with FVII deficiency cannot be reliably predicted; it may be related to the severity of trauma, the fibrinolytic activity of surgical sites, or a compensatory increase in fibrinolysis system activity compared to healthy individuals. However, further experimental evidence is needed to support these hypotheses.

## Data Availability

The original contributions presented in the study are included in the article/supplementary material, further inquiries can be directed to the corresponding author.
